# Patient Experiences and Preliminary Effects of Virtual Reality Based Pain Treatment in Rheumatoid Arthritis Patients With Persistent Pain Despite Low Disease Activity: A Mixed Method Pilot Study

**DOI:** 10.1002/msc.70251

**Published:** 2026-07-29

**Authors:** Stijn J. M. Temmink, Marlou A. Bakker, Demy D. Gerritsen, Mirjam C. Hegeman, Liza de Vries, Peter M. ten Klooster, Christina Bode, Harald E. Vonkeman

**Affiliations:** ^1^ Department of Psychology Health and Technology University of Twente Enschede the Netherlands; ^2^ Department of Rheumatology and Clinical Immunology Medisch Spectrum Twente Enschede the Netherlands

**Keywords:** pain management, persistent pain, rheumatoid arthritis, virtual reality

## Abstract

**Objective:**

This study aimed to examine the acceptability, usability, patient perceptions and preliminary effects of a virtual reality (VR) based pain management programme for people with rheumatoid arthritis with persistent pain despite low disease activity.

**Methods:**

This study used a two‐phase mixed‐methods approach with questionnaires and qualitative interviews. During the first phase of this study, 21 participants completed the virtual reality sickness questionnaire (VRSQ) and a 0‐10 NRS pain before and after using the VR programme once. Semi‐structured individual interviews were conducted on the perceptions of patients. In the second phase of the study, five participants used the VR programme for 8 weeks at home. Participants answered a NRS pain twice a week and the VRSQ, ASES and SF‐36 before and after the experiment.

**Results:**

In the first phase, a clinically meaningful reduction in pain intensity was observed in 10% of participants after one session of VR. Virtual reality sickness symptoms worsened for some and improved in others. Participants found the programme usable. Patients perceived relaxing and distracting effects. In the second phase, the median NRS pain intensity decreased by one point. Quality of life appeared to improve in the physical domain, whereas self‐efficacy remained stable. Virtual reality sickness symptoms remained stable or showed slight improvements overall.

**Conclusion:**

The VR‐based pain management programme was considered acceptable and usable by most people with RA. The programme also tended to slightly reduce pain. A larger randomised controlled trial is needed to determine the effectiveness of the programme.

## 1 Introduction

Rheumatoid arthritis (RA) affects approximately 0.5% of the general population leading to a lower quality of life due to persistent pain, which is the primary reason for visiting a rheumatologist (Heiberg et al. 2005; Ten Klooster et al. 2007; Venetsanopoulou et al. 2023). Treatment of RA consists of disease‐modifying anti‐rheumatic drugs (DMARDs), which ameliorate joint inflammation, ultimately resulting in lower pain (Ben Mrid et al. 2022). However, approximately 20% of people with RA who have achieved lower disease activity through DMARDs still suffer from persistent pain (Svensson et al. 2020; Ten Klooster et al. 2019). One mechanism for this disparity is that such patients may have developed nociplastic pain, which exists in the absence of tissue damage and results from altered processing in neural pain pathways, causing increased sensitivity to stimuli, also known as central sensitisation (Fitzcharles et al. 2021; Nijs et al. 2023). Moreover, people with RA with lower disease activity but persistent pain have been shown to experience hypersensitivity, hyperalgesia and allodynia, which are manifestations associated with nociplastic pain (Fitzcharles et al. 2021; Jansen, ten Klooster, Vonkeman, van den Berg, et al. 2023; Meeus et al. 2012).

Since medication does not always effectively reduce the pain experienced by these people with RA, non‐pharmacological solutions, such as physiotherapy and psychotherapy are used in the treatment of nociplastic pain (Bułdyś et al. 2023). However, access to these treatment options is limited due to their costs, the reliance on trained professionals and their time‐consuming nature (Becker et al. 2017; Driscoll et al. 2021). A possible solution may be the use of a virtual reality (VR) based pain management programme to treat nociplastic pain as it is more accessible via on‐demand usage and because it can integrate multiple current non‐pharmacological solutions (McGirt et al. 2023; O’Connor et al. 2022).

VR uses a headset that immerses users in a virtual three‐dimensional environment (Wohlgenannt et al. 2020). Due to the level of immersion and interaction, the user can feel present in a virtual world, leading to increased acceptance and effectiveness of the treatment (Mütterlein and Hess 2017; Pourmand et al. 2018). Currently, VR‐based non‐pharmacological treatment has been applied mostly for acute pain, where distraction is the main method of pain relief (Ahmadpour et al. 2019). For chronic pain, distraction, psychotherapy and education have been suggested as a way to restructure the neural pathways, leading to pain reduction in the long term (Bazzari and Bazzari 2022). One such VR application is Reducept (Reducept B.V. n.d.). This VR‐based pain management programme was developed for all types of chronic pain patients and has shown promising results among patients with chronic lower back pain (De Vries et al. 2023; Groenveld et al. 2023). The VR programme aims to reduce pain by accessing the hypothesised neuroplasticity of the brain to help rewire pain processing via combining distraction, pain education and principles from different psychotherapies (Reducept B.V. 2023).

As VR‐based pain management is a novel eHealth solution for reducing pain in people with RA, it is important to first investigate the acceptability and usability of this technology in this patient population (Van Gemert‐Pijnen et al. 2018). Acceptability is defined as the level to which the VR aligns with the user experience, for instance in terms of safety or emotions (Newton et al. 2021). Usability refers to the perceived ease of using the VR programme and headset (Newton et al. 2021). Moreover, although the aim of the VR‐based pain management programme is to rewire pain processing, little is known about the perceptions of people with RA with persistent pain on the value of VR. Additionally, preliminary effects in reducing symptoms should also be explored. Therefore, this study aims to explore the acceptability, usability, patient perceptions, and preliminary effects of VR‐based pain treatment in people with RA with persistent pain despite low disease activity. This study serves as a proof‐of‐concept pilot study.

## 2 Materials and Methods

To evaluate VR treatment for patients with RA suffering from persistent pain, a pilot study was conducted in two distinct phases, with the first studying the acceptability, usability, and patient perceptions of VR in a hospital environment and the second studying the acceptability and effects of utilising VR in a home environment.

### 2.1 Participants

Eligible participants were recruited from the rheumatology department at Medisch Spectrum Twente (MST) hospital, located in Enschede, the Netherlands. The inclusion criteria included age ≥ 18 years, a clinical diagnosis of rheumatoid arthritis with a disease duration ≥ 2 years, persistent pain ≥ 3 months, meaning two visual analogue scale (VAS) pain scores ≥ 4 within 3 months, and Dutch language proficiency. Exclusion criteria entailed no acute pain or intermittent pain complaints. Patients with severe audio‐visual complaints or comorbidities such as vertigo, dizziness, limited cognition, psychiatric history, vision complaints, and balance disorders were also excluded.

For the second phase of the study, additional criteria were implemented. Participants were required to have a VAS pain score in the past week ≥ 4. Furthermore, participants needed to have demonstrated stable low disease activity, defined as a Disease Activity Score (DAS28) < 3.2 at two separate time points at least 6 months apart, or DAS28 < 3.2 at one time point with the opinion of the rheumatologist of stable low disease activity, or a difference between tender joint counts and swollen joint count ≥ 4 at 2 or more moments at least 6 months apart. Additionally, participants had to have internet access to utilise the VR programme in a home setting. Ethical approval was appointed by the science commission of the science commission of the MST hospital.

### 2.2 Materials

The Oculus Go‐VR headset (Facebook Technologies, Menlo Park) was used with Reducept VR software version 1.3.4 (Reducept, Groningen, the Netherlands) during the first phase. For the second phase, the PICO G2 4K (PICO Interactive, Mountain View) was used with Reducept version 1.12.5. Both headsets are standalone VR headsets with integrated audio speakers, a high‐resolution screen, an integrated microphone, 360‐degree head‐tracking, and a virtual pointer controller.

For the first phase of the study, Reducept consisted of four exercises, each taking place in a virtual nerve system (Kuipers et al. 2023). First, the user enters the peripheral nerve system, where the user learns to regain control over pain by metaphorically neutralising painful stimuli. The next exercise takes place in the spinal cord, where cognitive behavioural therapy is applied, and users learn relaxation techniques. The third exercise visualises the brain and uses an exercise based on eye‐movement desensitisation and reprocessing mechanisms. Fourth, the user enters the alarm centre, where an acceptance and commitment therapy metaphor is used to focus on thoughts, feelings, and reactions to pain and how to deal with them. Prior to the second phase of the study, a new exercise was added to the Reducept application to educate and relax patients. This new fifth exercise visualises a control room and is designed to help patients stay attuned to their bodies and to release negative thoughts. Each exercise educates patients on the cause and effects of pain and has an approximate duration of 10 minutes.

Participants were asked to briefly review the Reducept manual prior to using the VR programme to facilitate easier navigation and understanding of the application's functionalities. For the first phase, data from the study questionnaires were collected using a tablet computer through the web‐based programme Qualtrics. Additionally, individual interviews were conducted through online video calls. For the second phase, during the baseline and follow‐up, Qualtrics was used to collect data. During the 8 weeks of the experiment, participants filled out questionnaires on paper.

#### 2.2.1 Outcome Measures

Acceptability was assessed in terms of potential side‐effects using the Virtual Reality Sickness Questionnaire (VRSQ) and user experience (UX) using a selection of subscales from the unified UX questionnaire (Kim et al. 2018; Tcha‐Tokey et al. 2016). The VRSQ uses 10 items (e.g., ‘To what extent do you currently experience general discomfort?’). The items were translated into Dutch and verified through a forward–backward translation with a bilingual native English speaker. From the unified UX questionnaire, the current study used the subscales of presence, engagement, immersion, flow, emotions, skill, and technology adoption. All items were answered on a 10‐point Likert scale and the subscales used showed acceptable reliability (Cronbach's alpha > 0.70) (Tcha‐Tokey et al. 2016).

The usability of Reducept was measured using an investigator‐developed questionnaire based on the unified UX questionnaire (Supporting Information S1: Appendix S1) (Tcha‐Tokey et al. 2016). The original questionnaire was adapted to emphasise the usability aspects of VR, since no validated survey captured the context of home‐based VR. This survey was intended as an exploratory measure and consisted of two open‐ended questions concerning ease of use of the VR programme and the VR headset, eight yes‐or‐no questions about usability, and four questions regarding clarity and difficulty of the programme. Prior to use, the questionnaire was reviewed by the research team to ensure face validity and relevance to the study aims.

A semi‐structured interview scheme was drawn up to gain a deeper understanding of the patients' experience using Reducept, its effectiveness and value as a pain management tool (Supporting Information S1: Appendix S2). The interview scheme consisted of 28 questions and was informed by the study objectives and existing literature on the topic. The research team created the interview scheme as an exploratory qualitative tool rather than a validated measurement instrument. An example question from the interviews is: ‘How did you experience the VR session?’ Additionally, the interviewer asked probes such as ‘Why not?’ to gain a deeper understanding of the participant's experience.

Effects were measured in terms of pain severity, health‐related quality of life (HRQoL) and self‐efficacy measured with a numerical rating scale (NRS), the 36‐item short‐form health survey (SF‐36) and the arthritis self‐efficacy scale (ASES), respectively (Hawker et al. 2011; Lorig et al. 1989; Ware 2000). The paper‐and‐pencil NRS was used for the question, ‘How much pain do you experience at this moment?’. The scale ranged from 0 to 10, where 0 represents ‘no pain at all’ and 10 represents ‘worst pain possible’. The SF‐36 measures HRQoL in eight different subscales, such as physical functioning, vitality as an indication of self‐reported fatigue, and general health. The Dutch SF‐36 has demonstrated adequate reliability and construct validity in people with RA (Ten Klooster et al. 2013). The ASES consists of 20 items divided into three categories: self‐efficacy in managing pain, physical function, and ability to control other symptoms. Items were rated on a four‐point Likert‐type scale. The reliability of the subscales in previous studies ranged from acceptable to good, with Cronbach's alpha ranging from 0.76 to 0.89 (Brady 2011).

The quality of the pain experience of the people with RA was assessed using the Generalised Pain Questionnaire (GPQ) (van Bemmel et al. 2019). It consists of seven items measuring symptoms of hypersensitivity often associated with nociplastic pain. The items were rated from 0 ‘never’ to 4 ‘very strongly’. The reliability of the questionnaire was rated good, with Cronbach's alpha being 0.82 (Jansen, ten Klooster, Vonkeman, and Buitenweg 2023).

### 2.3 Procedure

Participants meeting the inclusion criteria were invited to visit the hospital for the first phase of the study. Prior to starting the VR experiment, participants read and signed the informed consent form. Participants subsequently completed the sociodemographic and comorbidities questionnaires, VRSQ and NRS pain.

After familiarising themselves with the manual of the VR application, participants progressed through all four exercises of the programme in the presence of the researcher. After the intervention, participants again completed the VRSQ, the NRS pain, the selected subscales of the unified UX questionnaire and the self‐created usability survey. The whole experiment lasted approximately 1 hour.

Participants who completed all four exercises of the Reducept programme were invited to take part in a semi‐structured individual interview. This interview aimed to gain deeper insights into the patients' perceptions of the VR programme's impact on persistent pain.

In the second phase of the study, eligible participants were also invited to the hospital for their first appointment. After signing the informed consent form, participants completed the sociodemographic and comorbidities questionnaires, the VRSQ, GPQ, NRS, SF‐36, and the ASES. Participants then received instructions on using the VR headset and completed an introductory exercise in the VR programme. After completing the exercise, participants filled in the VRSQ again.

Participants were instructed to use the VR headset at home at least three times weekly for 10–15 minutes over an 8‐week period. Throughout these 8 weeks, they completed the NRS pain questionnaire twice a week. During the study, participants could contact the researcher via telephone or email with any questions or difficulties. In the first week, all participants were contacted by the researcher to ensure they were able to use the VR headset and to complete the NRS questionnaire. After 8 weeks, participants returned to the hospital for their end‐of‐study appointment. They used the VR headset once more and completed the VRSQ, SF‐36, and ASES questionnaires.

### 2.4 Analysis

For the statistical analysis, RStudio (version 2023.12.1+402) was used. Descriptive statistics were performed to summarise the participants' characteristics by calculating the median and interquartile range (IQR) based on distributional assessment using the Shapiro–Wilk test. Changes in NRS pain scores were assessed using the Wilcoxon signed‐rank test, and effect sizes were estimated using the Hodges–Lehmann estimator, providing a robust, median‐based estimate of the typical paired difference without assuming normality. Clinically meaningful improvement in pain was defined as a reduction of ≥ 2 points in the NRS. Given the small sample size and the exploratory nature of the study, no formal hypothesis testing was performed for the other outcome measures. Therefore, the outcomes of the SF‐36 and ASES were explored using visualisations and narratively described. The individual interviews were analysed using thematic analysis (Braun et al. 2019). This analysis started bottom‐up in which codes were added until no new codes emerged during the analysis of new data. Previous interviews were re‐analysed after each new code was added.

## 3 Results

### 3.1 Study Population

**FIGURE 1:**
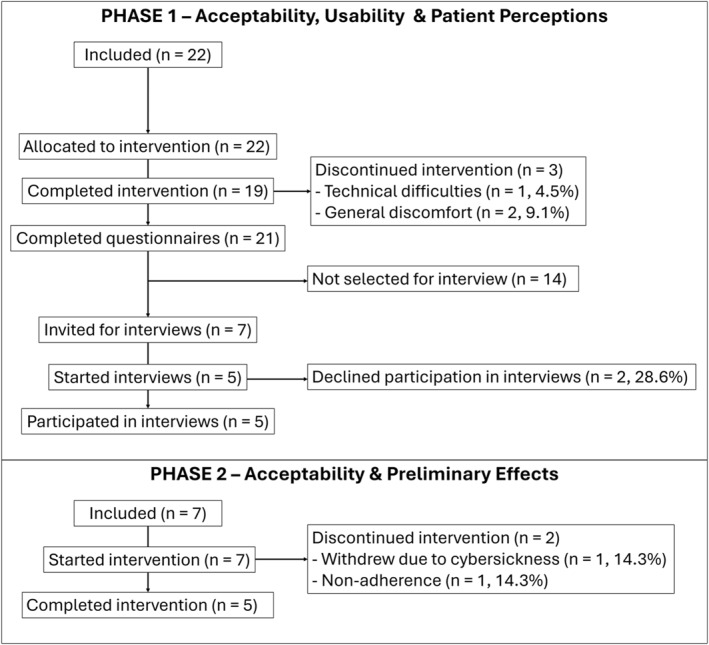
Flow diagram.

Phase 1 of the study enrolled 22 people with RA of whom 3 dropped out, one due to technical issues and two others due to general discomfort during VR use, but the latter two with sufficient use to continue answering the questionnaires (Figure 1). Participants' (*N* = 21) ages ranged from 40 to 85 years, 76% were female and the majority completed secondary education (57%) (Table 1). After the study, 7 participants were invited for an interview, to which 5 (4 females, 1 male) agreed.

**TABLE 1:** Demographic and clinical characteristics of the participants in phase one (*N* = 21).

Characteristic		
Sex	Male, *n* (%)	5 (24)
	Female, *n* (%)	16 (76)
Age group	40–59, *n* (%)	11 (52)
	60–79, *n* (%)	9 (43)
	80+, *n* (%)	1 (5)
NRS pain score at baseline	*Median* (*IQR*), range	6 (4–7), 2–8

The second phase of the study included 7 participants, 5 females and 2 males, with ages between 56 and 71 years (Table 2). Four of the participants used DMARDs in combination with NSAIDs, whereas the others only used DMARDs. Four participants scored > 10 on the GPQ, indicating hypersensitivity and nociplastic pain. Two participants suffered from balance problems, one from dizziness and another participant from vertigo. One of these participants dropped out of the study after the first appointment due to cybersickness. Another participant failed to use the VR headset at home and did not answer the twice‐weekly NRS questionnaires and thus was excluded from the final appointment. The other five participants completed the 8 weeks of the study.

**TABLE 2:** Demographic and clinical characteristics of the participants of phase two (*N* = 7).

Characteristic		
Sex	Male, *n* (%)	2 (29)
	Female, *n* (%)	5 (71)
Age group	50–59, *n* (%)	4 (57)
	60–69, *n* (%)	2 (29)
	70+, *n* (%)	1 (14)
Medication use	DMARD only, *n* (%)	3 (43)
	DMARD and NSAID, *n* (%)	4 (57)
Generalised pain questionnaire	< 10	3 (43)
	≥ 10	4 (57)
NRS pain score at baseline^a^	*Median* (*IQR*), range	5 (5–5), 4–7

^a^ For the NRS pain score, data were available for *N* = 5 participants, as only participants who completed the study had baseline pain scores recorded.

### 3.2 Acceptability

Acceptability, which was defined as the level to which the VR aligns with the user experience, was studied in phase one using the VRSQ. Prior to the intervention, participants mainly suffered from fatigue‐like symptoms; two reported severe fatigue and six reported moderate fatigue (Supporting Information S1: Appendix S3). Oculomotor symptoms were more prominent before the intervention than the disorientation symptoms. After the intervention, the oculomotor symptoms were still the most common symptoms. For some participants, symptoms deteriorated (e.g., participant nine indicated worse general discomfort, fatigue, and headache), while for others, symptoms improved (e.g., participant 18 improved on all symptoms). Overall, the intervention caused no increase in virtual reality sickness symptoms.

Participants reported more positive emotions, such as joy, than negative emotions, such as boredom and anxiety, when using the VR pain management programme (Supporting Information S1: Appendix S4). Enjoyment was rated moderately high by participants with 17 participants rating the intervention between 5 and 8. Boredom and anxiety from the experiment were rated with a score lower than 3 by most.

Flow, the degree to which the VR technology was able to absorb the user, was rated higher than 4 by all participants (Tcha‐Tokey et al. 2016). Engagement, also known as interactivity, was scored moderately high, but one participant scored 1. Presence, the perception of being in the virtual world, and immersion, the sense of being absorbed by the virtual environment, were rated most often with a score of 7 and 8, respectively (Tcha‐Tokey et al. 2016). Overall, the users rated the characteristics of the VR experience as moderately high.

Scores for technological adoption ranged from 1 to 10, with some stating that it would be very easy to learn how to use the VR intervention whereas others stated the opposite. For skill, defined as the ability to select objects in the virtual reality, 8 participants rated their ability as 10, while one participant rated their skill as 1. Results showed that the perceptions of technological skills varied even in this group of patients who signed up voluntarily to explore the VR programme.

In phase two of the study, acceptability in terms of virtual reality sickness symptoms was also good. One participant reported severe headache and difficulty to focus and moderate fullness of head, blurred vision, and vertigo. However, these symptoms were already present before the first use of VR and increased slightly after the first use but disappeared after 8 weeks. All other participants mostly expressed none to light symptoms (Supporting Information S1: Appendix S5).

### 3.3 Usability

Usability indicated the perceived ease of use of the VR programme and headset. One participant in the first phase of the study indicated that using the programme was not totally clear. This participant found it somewhat difficult to use the programme and the controls. The rest of the participants all found the instructions and programme at least somewhat clear and the programme and controls easy to use. Moreover, most participants found wearing and using the VR headset and the programme pleasant. Some participants found the headset too heavy and uncomfortable. Others mentioned that the programme itself was not pleasant due to annoying sounds. When asked about troubles with the VR headset and the programme, one participant mentioned having issues with putting on the VR headset. In total, 19 participants (90%) would recommend its use to others.

### 3.4 Perceptions of the Value of VR

In the interviews conducted after the first phase of the experiment, participants reported several perceived effects of the VR programme on different components of pain perception, such as coping, symptom control, sleep and self‐efficacy. All participants mentioned the relaxing nature of the VR programme and its ability to distract from current problems as a useful coping strategy (Table 3). Participants stated that repetition is a key component for VR to result in effective pain relief. The programme provided participants with the opportunity to reduce their own pain, which was normally impossible for them. Active participation in pain reduction also resulted in some participants becoming emotional, as they could express happiness, sadness, or frustration. Besides these affective responses to the VR programme, one participant perceived improved sleep after one session. Finally, participants stated that the VR programme helped them to gain more knowledge on the possibilities of dealing with and accepting their pain and limitations, suggesting increased self‐efficacy.

**TABLE 3:** The perceived value of VR according to interview (*N* = 5).

Codes (frequency)	Example quotes
Relaxation (14)	‘Because at some point, especially in the last part, you find yourself in a very relaxed phase … but then it was really very relaxing and that was really nice’.
Focus shifting/distraction (10)	‘You just take your thoughts off the present, out of what you're struggling with, and you know that is always good of course and to do it this way is nice’.
Raised awareness (9)	‘It's also very nice that you know how it works and what actually happens and the awareness of that’.
Emotions (7)	‘It made me positively emotional. The idea that you can shoot something that hurts, that hurts a lot with you. That was real, that was intense, yes’.
Actively involved in pain reduction (6)	‘Actively working on your pain, but also being able to attack your own pain again at that moment’.
Repetition (5)	‘This should actually happen to you daily or weekly, well this it just has to be something recurring, and not just when things go badly’.

### 3.5 Preliminary Effects

The NRS pain scores in Phase 1 were non‐normally distributed according to the Shapiro‐Wilk test (baseline: *W* = 0.90, *p* = 0.035; post‐intervention: *W* = 0.91, *p* = 0.047). Therefore, pain scores are presented as median, decreasing from 6 (IQR 4–7) at baseline to 5 (IQR 3–6) after the intervention. A Wilcoxon signed‐rank test indicated a statistically significant reduction in NRS pain scores over time (V = 0, *p* = 0.001). The Hodges–Lehmann estimator suggested a typical decrease in 1 point (HL = −1), indicating a downward shift in pain scores across participants. A clinically meaningful improvement in NRS was observed in 10% of participants.

**FIGURE 2:**
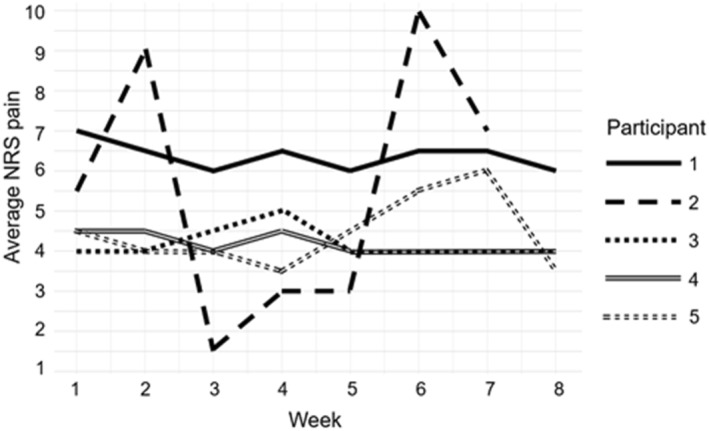
The course in NRS pain per participant.

In Phase 2, baseline NRS pain scores met the assumption of normality (*W* = 0.83, *p* = 0.135), whereas post‐intervention scores did not (*W* = 0.55, *p* = 0.001). Given the small sample size and the non‐normality of post‐intervention scores, data are presented as median (IQR). At the start of Phase 2 of the study, the median NRS pain score was 5 (IQR 5–5). After 8 weeks, this was slightly reduced to a median of 4 (IQR 4–4). A Wilcoxon signed‐rank test indicated a non‐significant reduction in NRS pain scores over time (V = 0, *p* = 0.072). The Hodges–Lehmann estimator indicated a 1 point decrease (HL = −1). The individual course of pain showed strong variations between participants (Figure 2). For instance, participants 1 and 4 showed stable pain scores, while participant 2 varied strongly, with scores fluctuating from 1 to 10.

**FIGURE 3:**
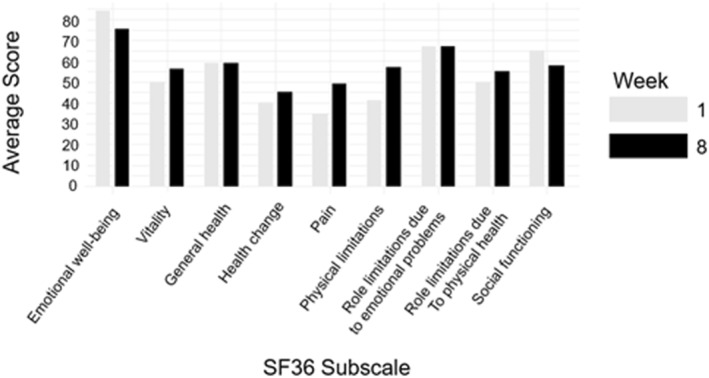
The average score on the subscales of the SF‐36 before and after the experiment. Grey indicates scores before using the VR pain management program, and black indicates scores after using the VR pain management program.

The SF‐36 subscales of vitality, health change, pain, physical function, and physical role limitations tended to improve over the 8 weeks of the intervention (Figure 3). The subscales general health and emotional role limitations remained the same, while the subscales emotional wellbeing and social functioning worsened. Thus, physical aspects of HRQoL, such as pain and physical functioning, mainly appeared to improve, whereas the psychological aspects, such as emotional well‐being and social functioning, remained the same or worsened after the use of VR.

**FIGURE 4:**
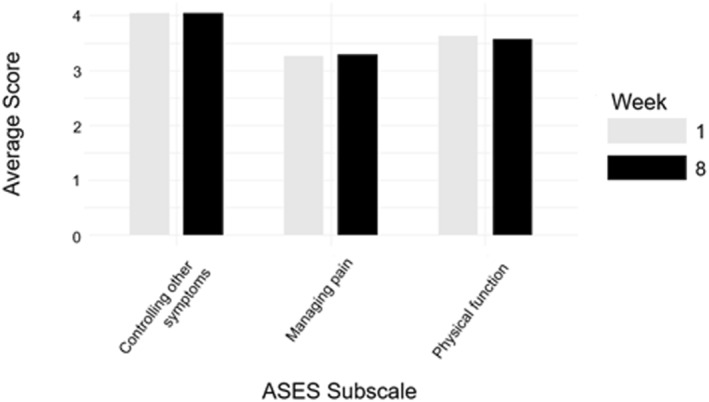
The average score on the subscales of the ASES before and after the experiment. Grey indicates scores before using the VR pain management program, and black indicates scores after using the VR pain management program.

In the second phase of the study, participants perceived their ability to manage their pain to be lower than their ability to function physically or to control other symptoms. This had not changed after 8 weeks (Figure 4).

## 4 Discussion

The aim of this study was to explore the acceptability, usability, patient perceptions on the value of VR and preliminary effects of a VR‐based pain management programme for people with RA with persistent pain despite low disease activity. Acceptability was relatively high with VR sickness symptoms not severely worsening after single use and at most moderately after 8 weeks. Usability of the VR headset was scored generally high, with most participants recommending it to others. Relaxation and distraction were most often mentioned as the added value of VR in pain management. VR was associated with small reductions in pain intensity after single and prolonged usage, with a substantial variation between participants. HRQoL tended to increase in the physical subscales, while remaining stable or showing some decrease in other aspects. Self‐efficacy remained unchanged.

VR sickness symptoms were rated as none to moderate after 8 weeks of exposure, which is in line with previous studies showing no severe symptoms after a longer period of using VR (Matthie et al. 2024; Stamm and Dahms 2023). Fatigue was the most mentioned and highest rated VR sickness symptom. While this could be due to the VR experiment, fatigue was often already rated high before using VR and could thus also be due to the vicious cycle between pain and fatigue, in which pain leads to more fatigue and vice versa (Løke et al. 2022; Matcham et al. 2015). Although low VR sickness symptoms were reported by participants who completed the study, a substantial drop‐out rate was observed, with two out of seven participants withdrawing in the second phase. This raises concerns regarding acceptability and potential selection bias towards more VR‐tolerant participants. Nevertheless, as a proof‐of‐concept pilot study, these findings highlight important practical challenges that must be addressed in future trials, such as improved screening for VR intolerance.

The proposed working mechanism of the VR‐based pain management programme is to use the neuroplasticity to rewire pain processing. Meanwhile, in the interviews of our study, participants mainly perceived the added value of VR to be relaxation and distraction. As the participants who were interviewed about their perceptions only used the VR programme once, it could be that only a short‐term distraction‐based working mechanism was noticed (Austin 2022). Furthermore, neuroplasticity as a mechanism is not noticeable by the participants and thus, should probably not be assessed via interviews but through imaging of the brain (e.g., fMRIs or QST) (Cheung et al. 2014; Georgopoulos et al. 2019). Understanding patient perceptions on the value of VR remains beneficial as it helps understand factors contributing to the acceptance and use of this pain management tool.

The pain intensity over the course of using the VR programme for 8 weeks varied greatly between participants and between measurements. The small sample size, combined with the large fluctuations in pain intensity observed in one participant may have influenced these results. However, De Vries et al. studied the same VR programme in chronic lower back pain and their results demonstrated less variation in pain intensity due to daily pain intensity reports (De Vries et al. 2023). These daily measures are influenced by circadian rhythm patterns of pain intensity in RA (Knezevic et al. 2023). Therefore, random measurements of pain intensity through the day are recommended. An additional advice for future studies is to measure pain intensity directly after the use of VR to reduce the effects of confounding factors.

The results showed that VR was associated with pain intensity reductions in a hospital setting and a home environment, which reinforces its potential as a self‐management tool. However, since the VR programme is a self‐management tool, self‐efficacy was hypothesised to increase after participation, which was not observed. A possible explanation is that the ASES captures disease‐related self‐efficacy, whereas participants in the interviews mainly described improved perceived coping during VR use, reflecting situation‐specific relaxation. Moreover, the ASES might not be sensitive to changes in self‐efficacy when using a technological intervention as users should be capable of the technology before they are able to lower their arthritis symptoms. The technological skills of participants varied and their technological self‐efficacy might have needed to increase before arthritis self‐efficacy could be increased. However, cognitive symptoms commonly reported by people with RA, including fatigue and brain fog, may make learning and adopting a novel VR‐based intervention more challenging for some participants, potentially contributing to the variability observed in usability perceptions and engagement with the intervention.

Although the reduction in pain intensity was small, the findings should be interpreted in the broader context of quality of life and pain‐related psychological factors. Indicated improvements in physical domains of the HRQoL suggest that VR may have contributed to better perceived physical functioning, even in the absence of large changes in pain intensity. In addition, self‐efficacy remained stable, indicating that patients did not experience increased confidence in managing their symptoms despite repeated VR use. However, qualitative findings suggest that participants experienced increased relaxation, distraction, and a greater sense of active involvement in pain management, which may reflect short‐term reductions in pain catastrophizing and improved coping. Together, these findings suggest that the VR intervention may primarily influence affective and cognitive aspects of pain rather than self‐efficacy or global pain control.

### 4.1 Limitations

The sample size in both phases of the study was small with a relatively high dropout rate, which limits the external validity and generalisability. The findings are further limited to patients with rheumatoid arthritis experiencing persistent pain despite low disease activity and may not be generalisable to other chronic pain populations. As a preliminary study, the findings are intended to inform future adequately powered randomised controlled trials. In addition, the study employed a single‐arm design without a control group, which limits causal inference regarding the observed effects. Selection bias may also be present as participation was voluntary and may have attracted individuals with greater interest or motivation toward VR‐based interventions. Prior experiences with VR among participants were not assessed, which may have influenced their perception of usability and acceptability. Furthermore, neither participants nor researchers were blinded, which may have introduced expectation bias in outcome reporting and observer bias in qualitative interviews. During the two phases of the study, different VR headsets and versions of the VR programme were used. While the objective of the intervention remained unchanged, the potential influence of these differences in outcomes cannot be fully excluded. Adherence to the prescribed VR usage was also not recorded, which limits insights into intervention engagement. This variability may have reduced the pain reducing effects of the VR intervention. VAS was used as an inclusion criterion as it is the standard practice in Dutch healthcare for recording pain intensity. However, this scale is not indicative of nociplastic pain, the target phenotype for this intervention. Future studies could instead use the Central Sensitisation Inventory or the Widespread Pain Index to include only RA patients with nociplastic pain. In addition, potential differences in pain phenotype and related symptom profiles, such as balance‐related complaints, were not systematically assessed, limiting the evaluation of their influence on treatment response and dropout. Moreover, usability was studied with an investigator‐developed, non‐validated questionnaire. Therefore, these findings should be interpreted as exploratory and not directly comparable to studies using validated usability instruments. Lastly, pain intensity was measured twice a week in the second phase, which may have reduced measurement accuracy. Future randomised controlled trials should consider these limitations when designing an optimised study protocol.

## 5 Conclusion

Based on this preliminary study, the VR‐based pain management programme was found to be acceptable and usable for most of the people with RA with persistent pain; however, VR intolerance may limit acceptability for a subset of patients and should be accounted for in future study designs. The perceived added value of the VR programme was relaxation and distraction. Effects in terms of pain intensity reduction were small and variable between participants and over time. To assess the effectiveness of this VR‐based pain management programme for people with RA with persistent pain, a large‐scale randomised controlled trial is needed. The results of this pilot study serve as a preliminary basis to inform future trials to be adequately powered.

## Author Contributions

H.V., C.B., and P.K. conceived and designed the study. H.V. was responsible for data collection. S.T. conducted the formal analysis. S.T. and M.B. draughted the original manuscript. All authors (S.T., M.B., D.G., M.H., L.V., P.K., C.B., and H.V.) critically revised the manuscript for important intellectual content, approved the final version of the manuscript, and agreed to be accountable for all aspects of the work.

## Funding

The authors have nothing to report.

## Conflicts of Interest

H.V. received unrestricted research grants and/or personal payments from Galapagos, Boehringer Ingelheim, AbbVie, Novartis, Pfizer, UCB, Janssen, Lilly, AstraZeneca.

## Supporting information


Supporting Information S1

## Data Availability

The data that support the findings of this study are available on request from the corresponding author. The data are not publicly available due to privacy or ethical restrictions.
