# Supplementary material for: Patient Experiences and Preliminary Effects of Virtual Reality Based Pain Treatment in Rheumatoid Arthritis Patients With Persistent Pain Despite Low Disease Activity: A Mixed Method Pilot Study
Source: Musculoskeletal Care. 2026 Jul 29;24(3):e70251. doi: 10.1002/msc.70251 (PMC13420965; doi:10.1002/msc.70251)
Supplement: Supplementary file 1 — Supporting Information S1 [file MSC-24-e70251-s001.docx]

**Appendices**

**Appendix 1. Self-created questionnaire regarding usability.**

During the Virtual Reality experience you used the program ‘Reducept’. The next questions regarding the use of ‘Reducept’ and/or the VR headset.

1. Did you enjoy using the VR headset?

○ Yes ○ No, because …………………

1. Did you enjoy using ‘Reducept?

○ Yes ○ No, because …………………

1. Was the VR headset easy to wear?

○ Yes ○ No, because …………………

1. Would you recommend ‘Reducept’ to others?

○ Yes ○ No, because …………………

Please indicate to what extent the following statements apply to you.

1. How clear was the explanation?

○ Very unclear ○ Somewhat unclear ○Somewhat clear ○Very clear

1. Was it clear what you should do when you had to use the program?

○ Very unclear ○ Somewhat unclear ○Somewhat clear ○Very clear

Please indicate to what extent the following statements apply to you.

1. How difficult did find it to use the VR headset and program?

○ Very difficult ○ Somewhat difficult ○Somewhat easy ○Very easy

1. How easy/difficult was it to control the VR headset and program?

○ Very difficult ○ Somewhat difficult ○Somewhat easy ○Very easy

1. What did you find easy about using the VR headset/program?

………………………………………………………………………………

1. What did you find difficult about using the VR headset/program?

………………………………………………………………………………

1. Did you experience problems with putting on and taking off the VR headset?

○ No ○ Yes, namely …………………

1. Did you experience problems with the controls of the program?

○ No ○ Yes, namely …………………

1. Did you experience other problems when using the VR headset/program?

○ No ○ Yes, namely …………………

1. Could you solve these problems independently?

○ Yes ○ No, I needed help with………

**Appendix 2. Semi-structured interview scheme regarding perceived working mechanism.**

*Introduction*

- Introducing researcher
- Thanking for participating in the research

You completed questionnaires before and after your VR session in the hospital. This questionnaires were mainly focused on the use of VR glasses. In this interview, I want you would like to ask some questions focused on your experiences and ideas regarding the effect of VR on chronic pain. Rheumatoid arthritis is associated with chronic pain complaints, even at times when there is no inflammation. Despite drug treatment, there is always a group of patients who still have pain complaints be a problem. This requires innovative treatment methods, including virtual reality could possibly be a part of. The precise working mechanism of VR on chronic pain is not yet known. The purpose of this interview is to gain more insight get into your ideas about the operation of VR and its application.

- I expect the interview to last approximately 30 minutes.
- After the last VR session in the hospital, you have already signed informed consent.
- Do you agree to record the interview so that I can retrieve it and myself listen and process the answers?
- The processing of the data is completely anonymous.
- There are no right or wrong answers, it is purely your opinion/idea.
- Do you have any questions in advance?

*General experience VR*

- What were your ideas/expectations prior to the VR session? Why?
- How did you experience the VR session? Can you tell us more about this?
- What makes it a positive/negative experience?

*Effectiveness*

You have now completed 1 VR session. To be effective for chronic pain in the long term

term, the advice of the makers of this program is to hold repeated sessions

found over a longer period of time.

- Has using VR given you any advantages/disadvantages? If so, how or what?
- Did you experience pain at the time of the VR session?
- Can you tell us something about the effectiveness of the session on your pain?
- Effect/reduction of pain: In what way? How do you think it worked? Can you give an example?

If there is no reduction: Why not?

In case of aggravation: What does that mean?

Imagine if you had 8 sessions instead of 1.

- With that idea in mind, what are your thoughts on the effectiveness of VR on pain?
- Why/why not?
- How often do you think it would be necessary to have an effect?
- Is this theoretically possible/feasible for you?
- How do you envision that? How often? How much time between sessions? (several times per day, every day, every other day, once a week, ...?) Where? Alone or accompanied?

*Exercises*

Nerve pathways – firing/restoring danger stimuli

- Do you remember this? What do you think was the effect/purpose of that?

Spinal cord – breathing exercise to close pain gates

- Do you remember this? What do you think was the effect/purpose of that?

Brain – reduce pain networks in the brain by re-creating connections

- Do you remember this? What do you think was the effect/purpose of that?

Pain alarm centre – dismiss danger, make alarm centre less sensitive to pain

- Do you remember this? What do you think was the effect/purpose of that?

Control room – mindfulness exercises focused on thoughts, breathing and the body

- Do you remember this? What do you think was the effect/purpose of that?

*Mechanisms*

The makers of the program have included various parts of which they expect that it may have an effect on the pain. One of those parts is:

Distraction

- Do you recognize this? How did you experience that? How do you think this will work? How do you think this could have helped?

Relaxation

- Do you recognize this? How did you experience that? How do you think this will work? How do you think this could have helped?

Education

- Do you recognize this? How did you experience that? How do you think this will work? How do you think this could have helped?

Gain more control over pain

- Do you recognize this? How did you experience that? How do you think this will work? How do you think this could have helped?

These are the 4 major parts that apply according to the makers.

- Do you have other ideas about this? If so, what? Why?
- Are there any other ways you think it has worked/could work?
- Are there other aspects that made you experience less pain?

*Tips for use*

- How do you think VR can/should be used in the future?
- What would make you not want to use VR in the future? Why?
- What would make you want to use VR in the future? Why?

*Ending*

- Do you have any other thoughts or additions/comments about the session?
- Do you have any questions?

Thank you for your cooperation. The data is processed anonymously. We hope with this to form a better picture of how VR can/will work for patients with RA and chronic pain. In the next stage of the research we will look at the effectiveness of repeated VR sessions on pain. If you are interested in the results of this If you have any research, please leave your (email) address with me and we will send you a summary of the results at the end of the project.

**Appendix 3. Heatmap showing the degree of virtual reality sickness symptoms.**
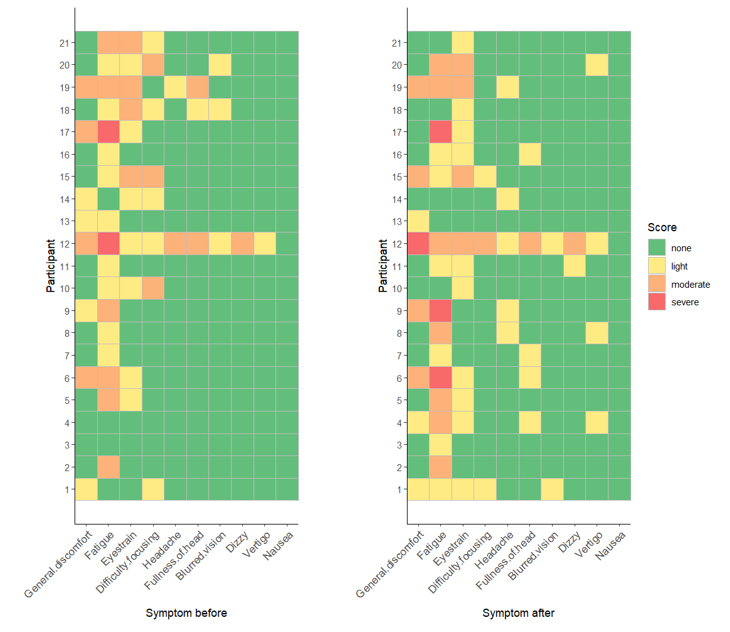
Note: Green indicates a score of 1 (none), yellow indicates a score of 2 (light), orange indicates a score of 3 (moderate) and red indicates a score of 4 (severe)

**Appendix 4. Heatmap of the average scores on the subscales of the unified UX questionnaire.**


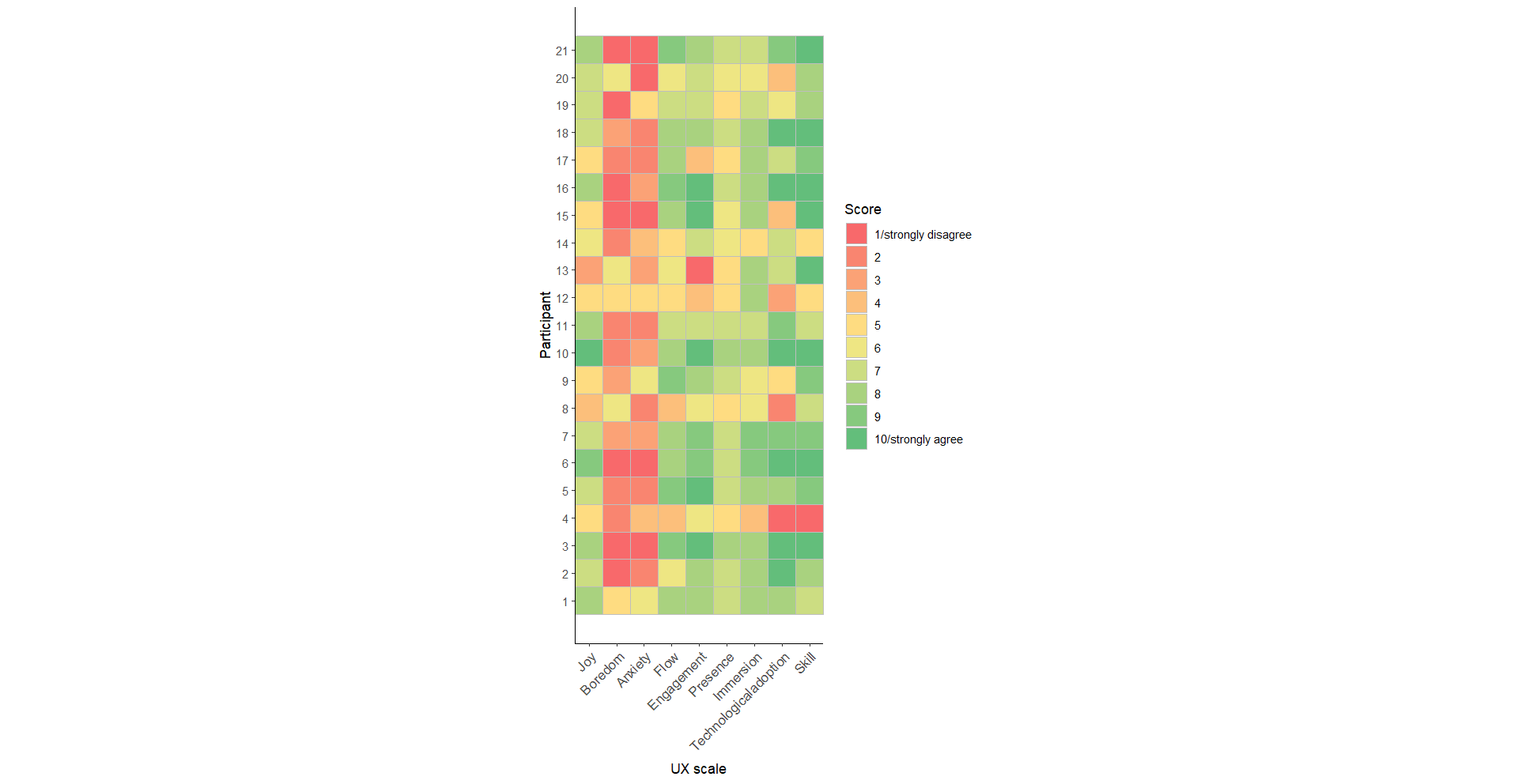


Note: The darkest red indicates a score of 1 (strongly disagree) and the darkest green indicates a score of 10 (strongly agree).

**Appendix 5. Heatmap of the degree of virtual reality sickness symptoms.**
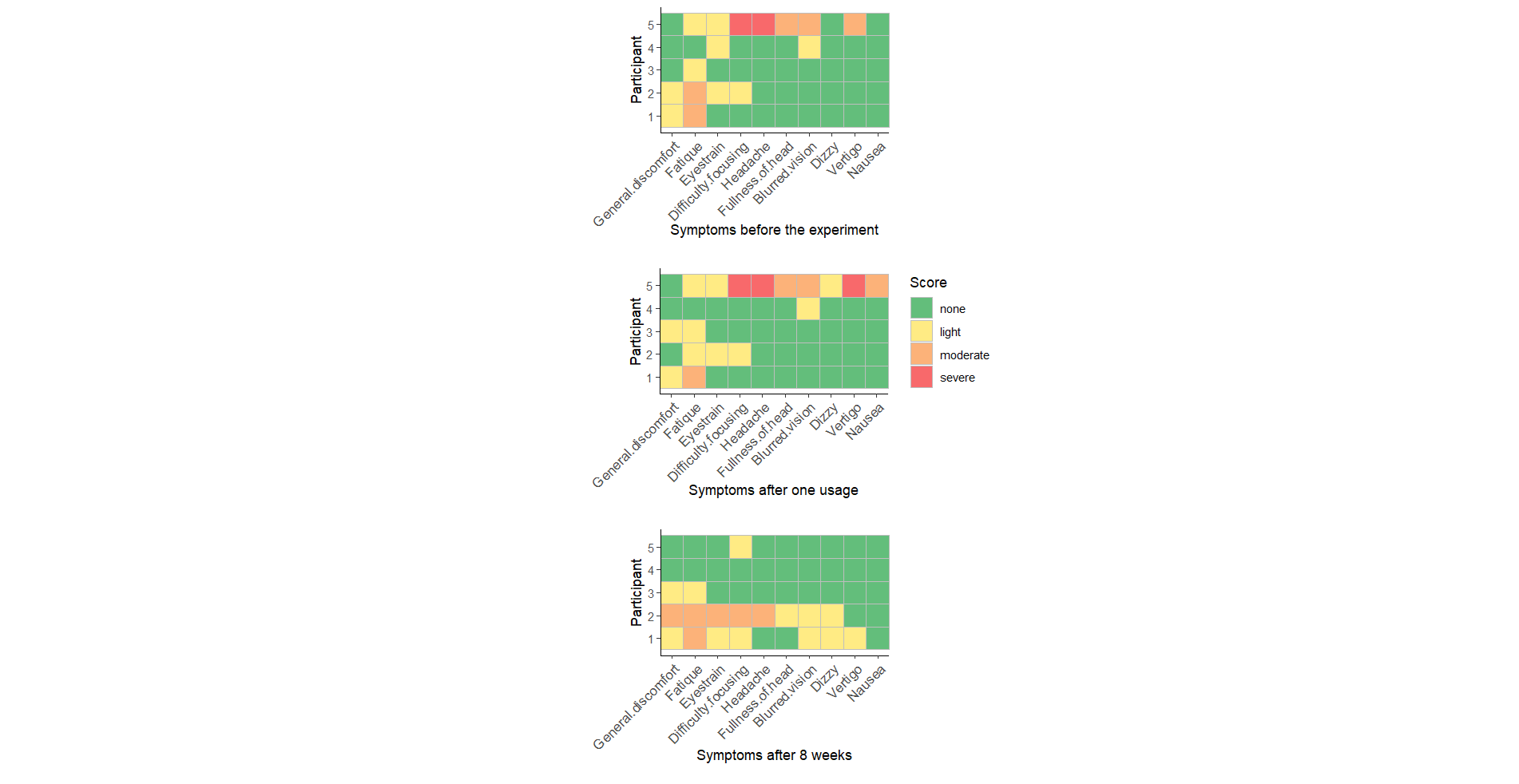


Note: Green indicates a score of 1 (none), yellow indicates a score of 2 (light), orange indicates a score of 3 (moderate) and red indicates a score of 4 (severe)
